# Association of perinatal sentinel events, placental pathology and cerebral MRI in neonates with hypoxic-ischemic encephalopathy receiving therapeutic hypothermia

**DOI:** 10.1038/s41372-022-01356-y

**Published:** 2022-02-28

**Authors:** Lia Hellwig, Muriel Brada, Ulrike Held, Cornelia Hagmann, Peter Bode, Karl Frontzek, Bernhard Frey, Barbara Brotschi, Beate Grass

**Affiliations:** 1 Division of Neonatology and Pediatric Intensive Care, University Children’s Hospital Zurich, Steinwiesstrasse 75, 8032 Zurich, Switzerland; 2 University of Zurich, Raemistrasse 71, 8006 Zurich, Switzerland; 3 Department of Pathology and Molecular Pathology, University Hospital Zurich, Raemistrasse 100, 8091 Zurich, Switzerland; 4 Epidemiology, Biostatistics and Prevention Institute, Department of Biostatistics, University of Zurich, Hirschengraben 84, 8001 Zurich, Switzerland; 5 Children’s Research Center, University Children’s Hospital Zurich, Zurich, Switzerland; 6 Institute of Neuropathology, University Hospital Zurich, Schmelzbergstrasse 12, 8091 Zurich, Switzerland

**Keywords:** Brain injuries, Pathogenesis

## Abstract

**Objective:**

Placental pathology might provide information on the etiology of hypoxic-ischemic encephalopathy (HIE). To evaluate the association of perinatal sentinel events (PSE), placental pathology and cerebral MRI in cooled neonates with moderate/severe HIE.

**Study design:**

Retrospective analysis of 52 neonates with HIE registered in the Swiss National Asphyxia and Cooling Register 2011–2019. PSE and Non-PSE groups were tested for association with placental pathology. Placental pathology categories were correlated with MRI scores.

**Results:**

In total, 14/52 neonates (27%) had a PSE, 38 neonates (73%) did not have a PSE. There was no evidence for an association of occurrence of PSE and placental pathologies (*p* = 0.364). Neonates with high MRI scores tended to have more often chronic pathologies in their placentas than acute pathologies or normal placentas (*p* = 0.067).

**Conclusion:**

Independent of the occurrence of PSE, chronic placental pathologies might be associated with more severe brain injury and needs further study.

## Introduction

Hypoxic-ischemic encephalopathy (HIE) affects 1-8/1000 term or near-term neonates and is a major cause of perinatally acquired neurological disabilities [1]. Limited placental blood flow leads to impaired gas exchange causing systemic acidosis and cerebral dysfunction that manifests as depressed consciousness and respiration as well as abnormal muscular tone [2]. Therapeutic hypothermia (TH) improves survival and neurodevelopmental impairment in neonates with moderate to severe HIE [3–5]. However, despite TH more than one third of all neonates with HIE show sequelae in their future life [3, 6].

Brain injuries following HIE can be detected with cerebral magnetic resonance imaging [7]. Injury to basal ganglia and thalamus has been associated with acute hypoxia-ischemia during a perinatal sentinel event (PSE), an acute event during birth causing disruption in placental blood flow [7, 8]. In contrast, “prolonged partial asphyxia” (Non-PSE) has been linked with white matter injury [7].

In recent years, different pathologies on placental examination such as insertion anomalies, histologic placental abruption [9], fetal malperfusion [10, 11], chorioamnionitis [12] and chronic villitis of unknown etiology [13] were reported in association with HIE.

In absence of PSE, chronic hypoxia-ischemia is still often assumed to be the cause of HIE. However, often this assumption is made by missing indication of PSE and without placental examination. This patient collective is likely not homogenous in terms of causes and pathophysiological pathways. Therefore, a better understanding of the correlation between history (PSE vs. Non-PSE), placental pathology and severity of brain injury may help to generate further hypotheses how to differentiate this patient collective. This knowledge might help to explain why not all neonates respond favorably to TH. Neonates with PSE seem to have a good response to TH for neuroprotection since TH is achieved within the necessary therapeutic window [8, 14]. The importance of placental examination has been stressed before [15, 16] as placental pathology might also help to understand the cause and time of the event (i.e., PSE vs Non-PSE) leading to hypoxia-ischemia. However, since the establishment of TH as standard neuroprotective therapy for moderate to severe HIE, these correlations have been insufficiently examined. Additionally, in prior publications placental pathology and MRI findings were evaluated with either complex or non-validated scores individual to each study [7, 9, 10, 15, 17].

The rationale is that a better understanding of the correlation between history (PSE vs. Non-PSE), placental pathology and severity of brain injury in neonates with HIE might lead to a better understanding of HIE and generate further hypotheses about its different underlying pathophysiological pathways.

The objective is to evaluate the association of PSE, perinatal clinical variables, placental pathology and cerebral MRI in neonates with moderate to severe HIE receiving TH.

## Methods

### Study population and data collection

Data collection, evaluation and publication for this study was approved by the Swiss ethical committee of the Canton of Zurich (KEK-ZH Number 2020-00102).

This is a retrospective single center cohort study including term and near-term neonates (≥ 35 weeks gestational age) with HIE registered in and treated according to the Swiss National Asphyxia and Cooling Register [18, 19]. All of them were outborn neonates and admitted to the neonatal/pediatric intensive care unit at the University Children’s Hospital Zurich between January 2011 and December 2019 for TH due to moderate to severe HIE. During this time span, no new guidelines were implemented. Obstetrical and neonatal management remained unchanged. TH with whole-body cooling was initiated within 6 h of birth, targeting 33.0–34.0 °C core temperature and continued for 72 h.

### Inclusion and exclusion criteria

Neonates were eligible for this study if the placenta was available for histopathological examination and if they had undergone cerebral MRI within two weeks after birth. If death occurred prior to the MRI, MRI was replaced by cerebral pathology findings on autopsy. Neonates were excluded due to missing placental examination and missing cerebral MRI or autopsy. Selection bias was addressed by using strict inclusion and exclusion criteria.

### Variables

Maternal and neonatal demographics, perinatal data and clinical data were collected from the entries in the Swiss National Asphyxia and Cooling Register and amended by review of the maternal and neonatal charts as appropriate.

Sarnat and Thompson scoring was performed by a senior neonatal consultant on admission [20, 21]. HIE was defined as moderate to severe encephalopathy (Sarnat stage 2 or 3, Thompson score ≥7) AND two out of the following perinatal data: (1) Apgar score <5 at 10 min, (2) Resuscitation after birth required >10 min, (3) pH ≤7.00 within first hour of life, (4) Base deficit ≥16 mmol/l within first hour of life, (5) Lactate ≥12 mmol/l within first hour of life [18].

A PSE was defined as an acute event in the perinatal history possibly compromising placental blood flow, taking place perinatally or causing immediate delivery. These included shoulder dystocia/head entrapment, uterine rupture, placental abruption, and umbilical cord mishap (i.e., cord prolapse or cord knot) [8, 22].

Placental examination was conducted by two trained pathologists blinded to clinical data and the MRI diagnosis. Ambiguities were resolved in consensus between the two pathologists. Placentas were weighed without membranes and umbilical cord and weight was classified according to percentile as described in the Atlas of Nontumor Pathology by Kraus et al. [23]. Placental pathology was evaluated according to the consensus statement by the Amsterdam Placental Workshop Group [24] and reported in 9 categories according to the synoptic reporting system proposed by Turowski et al. [25] (Supplementary Table 1). In this study, chorioamnionitis, meconium phagocytosis, acute conditions of fetal (cord knot with fresh thrombosis) and maternal malperfusion (acute to subacute infarcts, acute vasculopathy of basal plate or placental disruptions) were considered as acute pathologies. Chronic conditions of maternal perfusion (old infarcts, fibrinous deposits, distal villous hypoplasia, small placentas), chronic fetal malperfusion (any organized thrombosis in fetal vessels, cord knot with organized thrombosis) and delayed villous maturation were considered as chronic pathologies. Subsequently the placentas were grouped as “normal”, “acute”, “chronic” and “acute and chronic” (if an acute pathology complicated an underlying chronic pathology). These placental pathologies were summarized into two placental pathology categories: “normal placentas and placentas with acute pathologies” and “placentas with chronic pathologies”, containing placentas grouped as “chronic” and “acute and chronic”. It is well described in literature that TH must be initiated early (i.e. soon after the hypoxic-ischemic event) to be effective [14, 26, 27]. Therefore, neonates presenting with placentas with chronic pathologies were expected to be less responsive to TH.

MRI was performed after cooling within 14 days after birth. The MRI modes included T1 and T2-weighted pictures, diffusion-weighted pictures as well as spectroscopy [28]. MRIs were assessed by a neonatologist with large expertise in neuroimaging according to the score proposed by Weeke and de Vries et al. [29] (Supplementary Table 2). Grey matter (GM) subscore corresponds to injury to basal ganglia and thalamus, white matter (WM) subscore to white matter injury. In the grey matter subscore, grey matter spectroscopy was included. To address observer bias the neonatal neurologist was blinded to clinical data and the placental pathology. High MRI scores were defined as GM score > 10, low MRI scores as GM score ≤10. This cutoff was based on suggested grey matter cutoffs for risk of adverse neurodevelopmental outcomes as described by Weeke and de Vries et al. [29].

If death occurred prior to MRI, cerebral pathology was used as substitute for the MRI scans. A blinded neuropathologist reviewed all standard cerebral autopsy specimen and classified them according to pathological standards [30] depending on grey matter or white matter injury.

In this study the occurrence of PSE was tested for association with underlying placental pathology (i.e. “normal”, “acute”, “chronic”, “acute on chronic”), with the summarized placental pathology category (i.e. “normal placentas and placentas with acute pathologies”, “placentas with chronic pathologies”) and with MRI score. Severity of brain injury on MRI was tested for association with placental pathology category in the PSE group and Non-PSE group, individually as well as in the whole study population.

#### Statistical methods

Descriptive statistics were applied to compare included and excluded neonates and perinatal clinical variables of PSE vs. Non-PSE cases.

For categorical variables, counts and percentages of the total number of neonates were summarized. Normally distributed continuous variables were summarized as means and standard deviation, non-normally distributed or ordinally scaled variables were summarized as medians and interquartile ranges.

Differences between the two patient groups were considered exploratory and were calculated using the Student’s t-test for continuous, normally distributed variables, Wilcoxon rank sum test for continuous, non-normally distributed variables as well as ordinal variables and Χ^2^ or Fisher exact test for categorical variables. Whenever X^2^ test would have been applied and expected cell count was less than 5, Fisher’s exact test was used instead.

Χ^2^ or Fisher’s exact test, respectively, were used to test for association of occurrence of PSE and underlying placental pathologies, of PSE and underlying placental pathology category and of PSE and MRI score.

The relationship between the white matter and grey matter MRI score in dependence of the placental pathology category was visualized with scatterplots. The correlation between white matter and grey matter score was calculated using the Spearman Rho correlation coefficient with 95% confidence interval.

MRI scores were categorized into high MRI scores defined as GM score >10 and low MRI scores as GM score ≤10. Χ^2^ or Fisher’s exact test was applied as appropriate to test for association between the MRI score and the placental pathology or placental pathology category. The comparison was made for PSE and Non-PSE groups individually, as well as for the overall study population.

An exploratory data analysis using the same statistical tests was conducted as the MRI data distribution optically suggested the division into a group with high and a group with low combined (i.e., gray and white matter) MRI scores. High combined MRI scores were defined as GM score > 10 and/or WM score ≥5. Low combined MRI scores were defined as GM score ≤10 and WM score <5.

All tests applied in this study were two-tailed and a significance level of 0.05 was used. All analyses were conducted using SPSS Statistics 26.

## Results

### Clinical variables

**Fig. 1:**
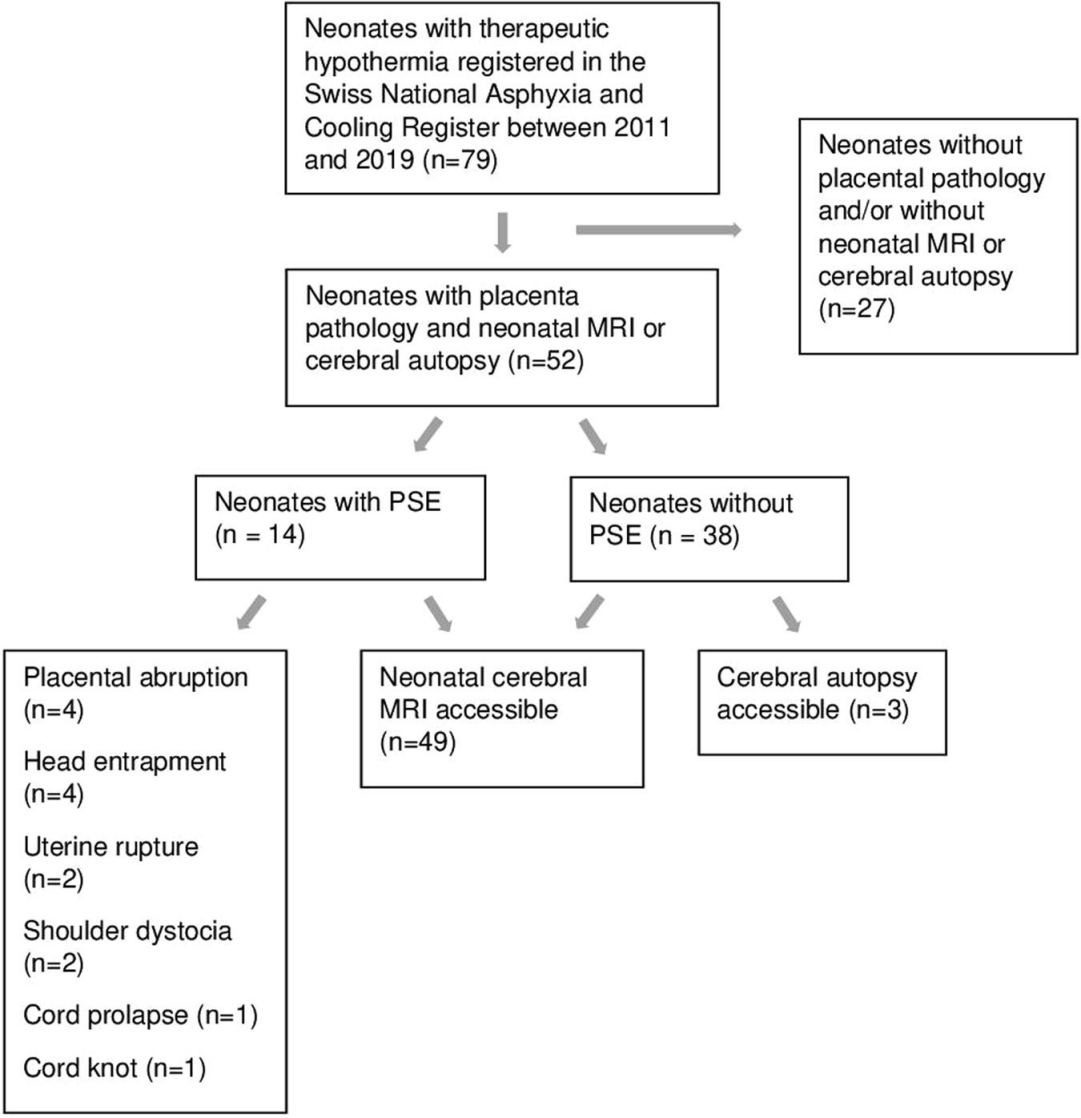
Flow chart of study population. Flow chart depicting included and excluded neonates as well as the availability of cerebral MRIs, placental pathologies and the occurrence of PSEs.

There were 79 neonates with HIE recorded in the Swiss National Asphyxia and Cooling Register between 2011 and 2019 who received TH in the neonatal/pediatric intensive care unit at the University Children’s Hospital Zurich. Demographics of 52 included and 27 excluded neonates were comparable (Supplementary Table 3), except for lower APGAR score at 1 min and higher mortality during TH in the excluded neonates (no MRI, no autopsy available), despite comparable severity of encephalopathy between groups. 49 of 52 neonates (94%) had neonatal MRI accessible, 3 of 52 neonates (6%) died before MRI was conducted and had cerebral autopsy available. In total, 14 of 52 neonates (27%) presented with a PSE, 38 of 52 (73%) had no indication of a PSE. Figure 1 shows the specific PSEs. Maternal illegal drug use, maternal seizures, preeclampsia, and placenta previa did not occur in this cohort. All included neonates were singleton pregnancies.

Perinatal clinical variables are shown in Table 1. There was no evidence for differences between the PSE and the Non-PSE group except for the mode of delivery (*p* = 0.018). Neonates with PSE were less often delivered by spontaneous vaginal delivery compared to neonates in the Non-PSE group (14% vs 56%).

**Table 1:** Perinatal clinical variables of the total study population and of the PSE and Non-PSE groups.

	Total (*n* = 52)	PSE (*n* = 14)	Non-PSE (*n* = 38)	*p* value
Gestational age (days, mean, SD)	279 (11)	280 (10)	279 (11)	0.745
Gender female (n, %)	24 (46.2%)	6 (42.9%)	18 (47.4%)	0.772
Birth weight (g, mean, SD)	3268 (466)	3460 (559)	3197 (413)	0.071
Head circumference (cm, mean, SD)	35.0 (1.9)	35.6 (1.5)^a^	34.7 (2.0)	0.172
Head circumference < 10^th^ percentile (n, %)	9 (17.6%)	1 (7.7%)^a^	8 (21.1%)	0.417
Placental weight (g, mean, SD)	429 (96)	430 (114)	428 (89)	0.952
Placental weight percentiles (Median, IQR)	25 (5-50)	15 (1-70)	25 (5-50)	0.825
APGAR 1 min (median, IQR)	2 (1–4)	1 (0-4)	3 (1–4)^a^	0.072
APGAR 5 min (median, IQR)	4 (2–6)	4 (2–5)^a^	4 (2–6)^a^	0.603
APGAR 10 min (median, IQR)	5 (3–6)	4 (3–6)^a^	5 (3–7)^a^	0.519
Resuscitation required > 10 min (n, %)	30 (57.7%)	9 (64.3%)	21 (55.3%)	0.559
Worst pH within 60 min (mean, SD)	6.9 (0.1)	6.8 (0.1)^a^	6.9 (0.2)^b^	0.493
Sarnat score on admission (median, IQR)	2 (2–3)	2 (2–3)	2 (IQR2-2)	0.300
*Sarnat 2 (n, %)*	37 (71.2%)	8 (57%)	29 (76%)	
*Sarnat 3 (n, %)*	15 (28.8%)	6 (43%)	9 (24%)	
Seizures (n, %)	13 (25%)	5 (35.7%)	8 (21.1%)	0.300
Death (n, %)	7 (13.5%)	1 (7.1%)	6 (15.8%)	0.659
*Died on day (median, IQR)*	6 (2–8)	6 (6-6)	6 (2–8)	
MRI accessible (n, %)	49 (94.2%)	14 (100%)	35 (92.1%)	0.555
MRI done on day (median, IQR)	6 (IQR 5–7)	6 (IQR 5–7)	6 (IQR 5–7)	0.624
Delivery mode (n, %)			^b^	0.018
SVD cephalic	22 (44.0%)	2 (14.3%)	20 (55.6%)	
Instrumental	10 (20.0%)	5 (35.7%)	5 (13.9%)	
Emergency CS	18 (36.0%)	7 (50.0%)	11 (30.6%)	
Pathological CTG (n, %)	22 (42.3%)	3 (21.4%)	19 (50%)	0.064
Increased risk of infection^c^ (n, %)	13 (25%)	4 (28.6%)	9 (23.7%)	0.729
Maternal diabetes (n, %)	5 (9.6%)	0 (0%)	5 (13.2%)	0.307
Time to reach target temperature (hours, mean, SD)	4.7 (2.8)	4.3 (1.7)	4.9 (3.1)	0.542

^a^Data was missing for one neonate in this group.

^b^Data was missing for two neonates in this group.

^c^Increased risk of infection: GBS positive mother, maternal fever under delivery, premature rupture of membranes (>18 h).

### Placental pathology

Placental pathologies with regard to occurrence of PSE are shown in Table 2 (further details: Supplementary Table 4). Out of 8 neonates with placentas classified as “acute”, one neonate from the Non-PSE group presented with histologic signs of placental abruption. This case was excluded from further analysis, as placental abruption was only diagnosed by placental examination and in this study, PSE was identified by history taking only.

**Table 2:** Placental pathology and occurrence of PSE.

Placental pathologies	PSE (*n* = 14)	Non-PSE (*n* = 38)
Normal	6	10
Acute	1	7
Chronic	6	12
Acute and chronic	1	9

There was no evidence for an association between the occurrence of PSE and placental pathologies (*p* = 0.364). When summarized into placental pathology categories, there was no evidence for an association between the occurrence of PSE and placental pathology category either (*p* = 0.736).

Of the three neonates whose cerebral MRI was replaced by cerebral autopsies, two presented with acute and chronic placental pathology, one neonate presented with chronic placental pathology.

### Cerebral MRI

Cerebral MRI was performed at a median age of 6 days (IQR 5–7).

**Fig. 2:**
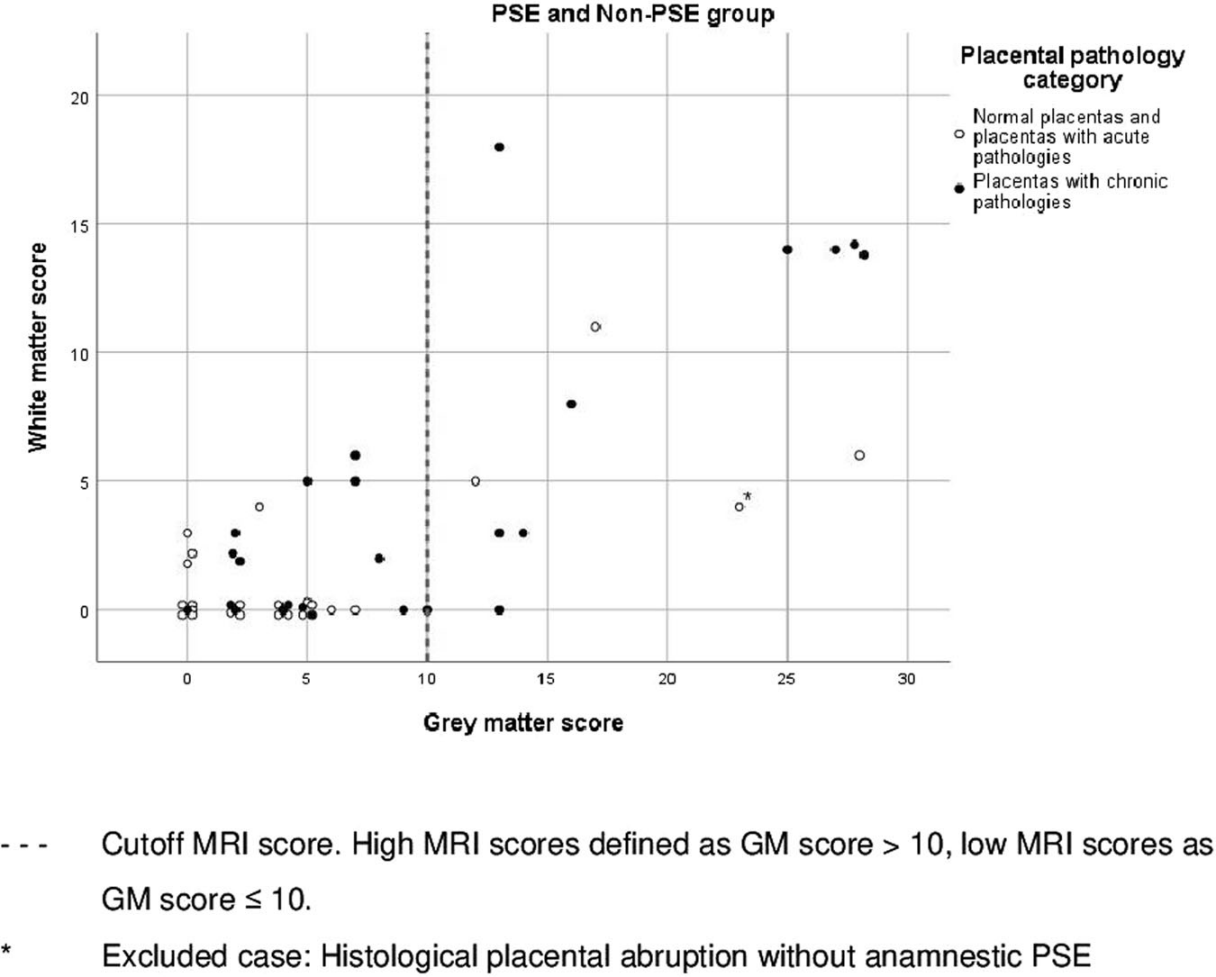
MRI scores and placental pathology category. Scatterplot depicting the distribution of white and grey matter scores of the whole study population according to placental pathology category.

Over the whole data set, there was a moderate positive correlation (Spearman Rho coefficient = 0.554, *p* < 0.01, 95% CI = 0.285–0.746) between white and grey matter subscore (Fig. 2). There was no evidence for an association between occurrence of PSE and MRI score (*p* = 1.00).

All three cerebral autopsies showed moderate to severe perinatal hypoxic-ischemic injury in predominantly grey matter areas. Two showed severe, one showed moderate injuries.

Neonates with more severe brain injury (i.e., higher MRI scores) tended to show chronic placental pathologies (75%, 9/12 neonates with high MRI scores), while neonates with less severe brain injury (i.e., lower MRI scores) more often presented with normal placentas or acute placenta pathologies (56%, 20/36 neonates with low MRI scores) (Fig. 2). This was seen in the PSE as well as in the Non-PSE group (Supplementary Fig. 1a, b).

However, there was no statistical evidence of an association between the placental pathology categories and severity of MRI scores. This is true for the overall study population (*p* = 0.067) as well as for the PSE and Non-PSE groups individually (*p* = 1.00 and *p* = 0.125, respectively).

An exploratory analysis was added since the MRI data distribution in Fig. 2 optically suggested an exploratory cutoff. The exploratory analysis provided evidence for an association of the placental pathology category and the combined MRI score in the overall study population (*p* = 0.009, OR 6.154; 95% CI 1.451–26.105). Neonates with high combined MRI scores tend to have chronic placental pathologies, whereas neonates with low combined MRI scores tend to present with normal placentas or acute placental pathologies (Fig. 2). This tendency was seen both in the PSE group and in the Non-PSE group (Supplementary Fig. 1a, b). However, there was no statistical evidence of an association between the placental pathology categories and severity of the combined MRI scores for the PSE and Non-PSE groups individually (*p* = 0.125 and *p* = 0.103, respectively).

## Discussion

In this study, the association between PSE, placental examination and severity of brain injury was investigated in a single center study including 52 cooled neonates with HIE registered in the Swiss National Asphyxia and Cooling register between 2011 and 2019.

The findings suggest an association between severity of brain injury and chronic placental pathologies, independent of evidence of PSE.

This is a hypothesis generating study with a small sample size. The correlation of PSE, results of placental examination and severity of brain injury in neonates with moderate to severe HIE undergoing TH might help to understand the underlying timing and pathways of hypoxic-ischemic injury. Placental examination has been suggested to be an important factor in identifying more homogenous subgroups of neonates suffering from HIE and in developing additional preventive strategies [15]. Preventive strategies might include obstetric assessment for placental risk factors, maybe affecting timing and mode of delivery, as well as tailored neuroprotective strategies in the affected neonate in light of specific injury mechanisms.

The study cohort was representative of all neonates with moderate to severe HIE receiving TH at the University Children’s Hospital Zurich. Perinatal clinical variables of PSE and Non-PSE neonates are comparable. The statistically significant difference in mode of delivery is explained by the fact that if a PSE is noticed by the obstetrician, delivery will be expedited, leading to more instrumentally assisted births or emergency cesarean sections.

An interesting finding is that Non-PSE neonates more often presented with head circumference <10th percentile than PSE neonates. Although not statistically relevant, the smaller head circumference might be caused by chronic hypoxic-ischemic conditions. While acute perinatal hypoxic-ischemic injury has been associated with secondary microcephaly, chronic injury has been associated with microcephaly present at birth [31–33].

The distributions of placental pathologies (“normal”, “acute”, “chronic” and “acute and chronic”) as well as the placental pathology categories (“normal placentas and placentas with acute pathologies” and “placentas with chronic pathologies”) were independent of the occurrence of PSE.

As shown in Table 2, only one third of neonates with moderate to severe HIE had normal findings on placental examination. More than half of the placentas revealed chronic pathologies, previously associated with increased risk for HIE [9, 11, 13].

Therefore, placental examination plays an important role in the comprehensive work-up of neonates with HIE.

The tendency for neonates with severe brain injury, i.e. high MRI scores, to present with chronic as well as chronic and acute placental pathologies was both visible in the overall study population and in the PSE and Non-PSE groups individually. Most likely due to small sample size, statistical significance was not reached for this association.

Neonates with PSE seem to have a better response to TH, showing more often normal MRI and better neurodevelopmental outcomes than neonates without PSE [14]. This was attributed to the fact that the TH can be established within 6 h after the hypoxic incident, which takes place perinatally. Hence, neuroprotective target temperature can be achieved within the therapeutic window. In the reported cohort, half of the neonates with PSE had chronic pathologies in their placental examination. Therefore, we hypothesize that brain injury might persist despite TH, as the chronic hypoxic-ischemic injury occurred too early to be improved by TH. This study was underpowered to test this association. We assume that due to this high proportion of chronic placental pathologies and because all neonates received TH, grey matter predominant injuries were not distinguished and correlated to the PSE group as in previous studies [7, 8]. The reported findings on the severity of brain injury reflected by the MRI score support a positive correlation between the grey and the white matter subscore, as it was described by Weeke and de Vries et al. [29].

The generalizability of our results is limited due to the small sample size from a single center study. Placental pathology was reported in 9 categories proposed by Turowski et al. [25] and subsequently condensed into 4 placental pathologies and 2 placental pathology categories due to small sample size. As the sample sizes of placental categories are still very small, conclusions must be drawn with caution. Furthermore, the results from the exploratory analysis are based on a cutoff, which was suggested based on visual data distribution. It needs further investigation and validation in a larger cohort. A strength of this study is the design with prospective data registry and the uniform application of existing treatment guidelines for TH. Furthermore, the datasets were complete due to the standardized entry into the Swiss National Asphyxia and Cooling Register. The pathologists and the neonatologist scoring the neuroimaging were blinded. Two pathologists reviewed the placental specimen independently whereas MRIs were scored by a single observer.

While PSEs as placental abruptions, uterine ruptures and cord complications are clinically well defined, shoulder dystocia presents with different severities depending on the necessity to apply first- or second-line obstetrical maneuvers for delivery. As a limitation of this study it should be noted that shoulder dystocia is a subjective diagnosis [34].

Another limitation consists of the fact that neuropathological standard samples might underrepresent cerebral regions where white matter injury is expected.

As stressed in prior studies [15, 17] placental examination is an important diagnostic tool in assessing the etiology of HIE and remains of importance in the era of TH. Placental pathology might help to investigate underlying timing of injury pathways in HIE and explain individual responses to TH, sometimes resulting in persistent brain injury despite TH. The findings presented in this study generate the hypothesis that chronic placental pathologies might be associated with more severe brain injury independent of occurrence of PSE. These findings need to be further investigated and validated in a larger cohort of neonates with HIE. If chronic placental pathology proofs to be a risk factor for a more severe MRI score, possibly indicating a worse neurodevelopmental outcome, thorough sonographic examination of the placenta could lead to improved management of neonates at risk. Postnatal placental examination is highly recommended in neonates with HIE.

## Supplementary information

Supplementary Information

## Data Availability

The datasets used and/or analyzed during the current study are available from the corresponding author on reasonable request.
