# Supplementary material for: Association of perinatal sentinel events, placental pathology and cerebral MRI in neonates with hypoxic-ischemic encephalopathy receiving therapeutic hypothermia
Source: J Perinatol. 2022 Feb 28;42(7):885–91. doi: 10.1038/s41372-022-01356-y (PMC9259485; doi:10.1038/s41372-022-01356-y)
Supplement: Supplementary file 1 — Supplementary Information [file 41372_2022_1356_MOESM1_ESM.docx]

**Supplemental Tables and Figures:**

**Supplemental Table 1: Placental categories according to Turowski et al.**

**Supplemental Table 2: MRI score according to Weeke et al.**

**Supplemental Table 3: Perinatal clinical variables of the included and excluded neonates**

**Supplemental Table 4: Detailed placental pathology and occurrence of PSE**

**Supplemental Figure 1a and 1b: Figure 1a: MRI scores and placental pathology category of the PSE group; Figure 1b: MRI scores and placental pathology category of the Non-PSE group**

**Supplemental Table 1: Placental categories according to Turowski et al. (25)**

| **Category** | **Pathological feature** |
| --- | --- |
| **1** | Normal morphology according to gestational age |
| **2** | Chorioamnionitis |
| **3** | Villitis and intervillositis |
| **4** | Maternal circulatory disorders (decidual vasculopathy) |
| **5** | Fetal circulatory disorders |
| **6** | Delayed villous maturation |
| **7** | Findings suggestive of genetic aberration |
| **8** | Implantation disorders |
| **9** | Other lesions |

Adapted from „A new, clinically oriented, unifying and simple placental classification system” by Turowski et al. (25)

**Supplemental Table 2: MRI Score according to Weeke et al. (29)**

| **Items** | **Sequence used to assess injury** | **Degree** |  |  |
| --- | --- | --- | --- | --- |
| **Grey matter** |  | **0** | **1** | **2** |
| **Thalamus abnormal SI or diffusion restriction** | T1/T2  DWI | No | Focal (<50%) | Extensive (≥50%) |
| Specify location |  |  | Unilateral | Bilateral |
| **Basal ganglia abnormal SI or diffusion restriction** | T1/T2  DWI | No | Focal (<50%) | Extensive (≥50%) |
| Specify location |  |  | Unilateral | Bilateral |
| **PLIC myelination or diffusion restriction** | T1/T2  DWI | Normal or no diffusion restriction | Equivocal/partially myelinated or partial (<50%) diffusion restriction | Absent myelination or extensive (≥50%) diffusion restriction |
| Specify location |  |  | Unilateral | Bilateral |
| **Brainstem (peduncles) abnormal SI or diffusion restriction** | T1/T2  DWI | No | Focal (<50%) | Extensive (≥50%) |
| Specify location |  |  | Unilateral | Bilateral |
| **Perirolandic cortex diffusion restriction** | DWI | No | Mild | Clear |
| Specify Location |  |  | Unilateral | Bilateral |
| **Hippocampus diffusion restriction** | DWI | No | Yes |  |
| Specify location |  |  | Unilateral | Bilateral |
| **Basal ganglia NAA** | ^1^H-MRS | Normal | Reduced |  |
| **Basal ganglia lactate** | ^1^H-MRS | Absent | Increased |  |
| **White Matter/Cortex** |  | **0** | **1** | **2** |
| **Cortex abnormal SI or diffusion restriction not being periorlandic cortex** | T1/T2  DWI | No | Focal (1 lobe) | Extensive (>1 lobe) |
| Specify location |  |  | Unilateral | Bilateral |
| **White matter increased SI or diffusion restriction not being PWML** | T1/T2  DWI | No | Focal (1 lobe) | Extensive (>1 lobe) |
| Specify location |  |  | Unilateral | Bilateral |
| **PWML** | T1/T1  DWI  SWI | No | < 6 | ≥ 6 |
| Specify location |  |  | Unilateral | Bilateral |
| **Hemorrage not being PWML** | T1/T2  SWI | No | Single hemorrhage < 1.5cm | ≥ 1.5 cm or multiple hemorrhages |
| Specify location |  |  | Unilateral | Bilateral |
| **Optic radiation diffusion restriction** | DWI | No | Mild | Clear |
| Specify location |  |  | Unilateral | Bilateral |

**Supplemental table 3: Perinatal clinical variables of the included and excluded neonates**

|  | **Included**  **(n=52)** | **Excluded**  **(n = 27)** | **Total**  **(n = 79)** | **p-Value** |
| --- | --- | --- | --- | --- |
| **PSE** (n, %) | 14 (26.9%) | 9 (33.3%) | 23 (29.1%) | 0.552 |
| **Gestational age**  (days, mean, SD) | 279 (11) | 275 (13) | 278 (12) | 0.161 |
| **Gender female**  (n, %) | 24 (46.2%) | 11 (40.7%) | 35 (44.3%) | 0.646 |
| **Birth weight**  (g, mean, SD) | 3268 (466) | 3314 (538) | 3284 (489) | 0.692 |
| **Head circumference** (cm, mean, SD) | 35.0 (1.9)^1^ | 35.0 (1.3)^8^ | 35.0 (1.7) | 0.999 |
| **Head circumference**  **< 10^th^ percentile**  (n, %) | 9 (17.6%)^1^ | 1 (5.3%)^8^ | 10 (14.3%) | 0.188 |
| **APGAR 1 min**  (median, IQR) | 2 (1-4)^1^ | 1 (0-2) | 1 (1-3) | 0.033 |
| **APGAR 5 min**  (median, IQR) | 4 (2-6)^2^ | 3 (2-5) | 4 (2-5) | 0.137 |
| **APGAR 10 min**  (median, IQR) | 5 (3-6)^2^ | 3 (2-6) | 4 (2-6) | 0.106 |
| **Resuscitation required > 10 min**  (n, %) | 30 (57.7%) | 18 (66.7%) | 48 (60.8%) | 0.438 |
| **Worst pH within 60 min**  (mean, SD) | 6.9 (0.1)^3^ | 6.8 (0.2)^2^ | 6.8 (0.2) | 0.708 |
| **Sarnat score on admission**  (median, IQR)  ***Sarnat 2*** *(n, %)*  ***Sarnat 3*** *(n, %)* | 2 (2-3)  *37 (71.2%)*  *15 (28.8%)* | 2 (2-3)  *16 (59.3%)*  *11 (40.7%)* | 2 (2-3)  *53 (67.1%)*  *26 (32.9%)* | 0.320 |
| **Seizures**  (n, %) | 13 (25%) | 9 (34.6%) | 22 (28.2%) | 0.374 |
| **Death** (n, %)  ***Died on day*** *(median, IQR)* | 7 (13.5%)  *6 (2-8)* | 9 (33.3%)  *3 (2-4)* | 16 (20.3%)  *3 (2-7)* | 0.037  *0.192* |
| **Cooling reached** | 5 (3)^3^ | 4 (2)^1^ | 5 (2) | 0.494 |
| **Delivery mode**  (n, %)  **- SVD cephalic**  **- Instrumental**  **- Emergency CS**  **- Elective CS** | ^2^  22 (44.0%)  10 (20.0%)  18 (36.0%)  0 (0%) | ^1^  7 (26.9 %)  7 (26.9%)  11(42.3%)  1 (3.8%) | 29 (38.2%)  17 (22.4%)  29 (38.2%)  1 (1.3%) | 0.291 |
| **Pathological CTG**  (n, %) | 22 (42.3%) | 13 (48.1%) | 35 (44.3%) | 0.641 |
| **Increased risk of infection**^0^  (n, %) | 13 (25%) | 6 (22.2%) | 19 (24.1%) | 0.784 |
| **Maternal diabetes**  (n, %) | 5 (9.6%) | 2 (7.4%) | 7 (8.9%) | 0.743 |

^0^ Increased risk of infection: Mother GBS positive, maternal fever under delivery, premature rupture of membranes (>18hours)

^1, 2, 3, 8^ Data was missing for one, two, three or eight neonates in the labelled group

**Supplemental table 4: Detailed placental pathology and occurrence of PSE**

| **Placental pathologies** | **PSE**  **(n = 14)** | **Non-PSE**  **(n = 38)** |
| --- | --- | --- |
| **Normal** | 6 | 10 |
| **Acute** | 1   - 1 Acute choriamnionitis | 7   - 3 Acute chorioamnionitis - 2 Acute meconium phagocytosis - 1 Acute chorioamnionitis and meconium phagocytosis - 1 Acute placental abruption |
| **Chronic** | 6   - 1 Chronic maternal malperfusion - 4 Delayed villous maturation - 1 Delayed villous maturation and maternal malperfusion | 12   - 6 Delayed villous maturation - 2 Delayed villous maturation and maternal malperfusion - 1 Delayed villous maturation and fetal malperfusion - 1 fetal malperfusion - 2 maternal malperfusion |
| **Acute and Chronic** | 1   - 1 Acute and chronic fetal malperfusion | 9   - 2 Acute and chronic maternal malperfusion - 3 Acute chorioamnionitis and delayed villous maturation - 1 Acute chorioamnionitis and chronic maternal malperfusion - 1 Acute and chronic fetal malperfusion - 1 Acute fetal malperfusion, delayed villous maturation, chronic villitis, implantation disorder - 1 Acute placental abruption and delayed villous maturation |

| **Corus callosum diffusion restriction** | DWI | No | Yes |  |
| --- | --- | --- | --- | --- |
| **Cerebellum** |  | **0** | **1** | **2** |
| **Cerebellum abnormal SI or diffusion restriction** | T1/T2  DWI | No | Focal (<0.5 cm) | Extensive (≥ 0.5 cm or multiple lesions |
| Specify location |  |  | Unilateral | Bilateral |
| **Cerebellar hemorrhage** | T1/T2  SWI | No | Single hemorrhage <0.5 cm | ≥ 0.5 cm or multiple hemorrhages |
| Specify location |  |  | Unilateral | Bilateral |
| **Additional** |  | **0** | **1** | **2** |
| **Intraventricular hemorrhage** | T1/T2  SWI | No | Yes |  |
| **Subdural hemorrhage** | T1/T2 | No | Yes |  |
| **Cerebral sinusvenous thrombosis** | T1/T2  MRV | No | Yes |  |

Adapted from “A Novel Magnetic Resonance Imaging Score Predicts Neurodevelopmental Outcome After Perinatal Asphyxia and Therapeutic Hypothermia” by Weeke et al. (29)

**Supplemental Figure 1a: MRI scores and placental pathology category of the PSE group**


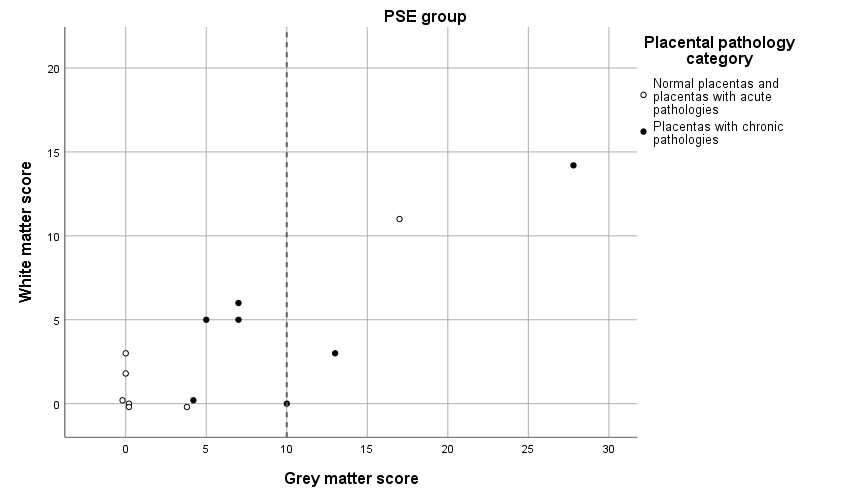


**Supplemental Figure 1b: MRI scores and placental pathology category of the Non-PSE group**


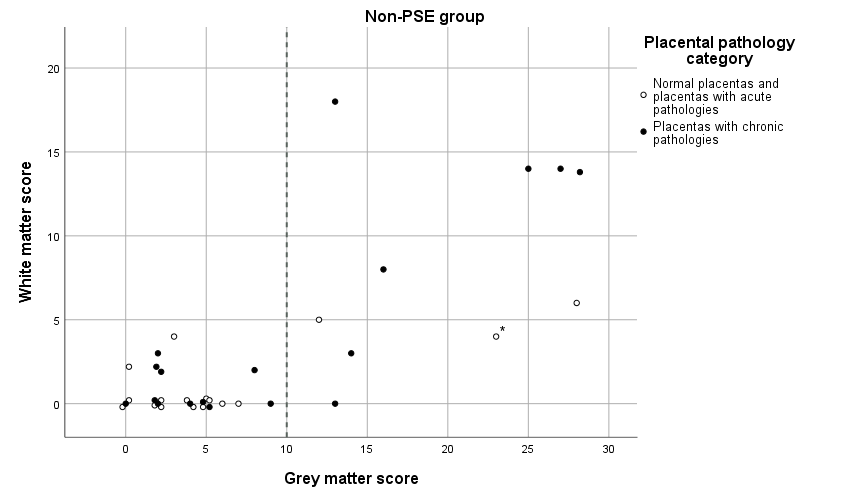


- - - Cutoff MRI score. High MRI scores defined as GM score > 10, low MRI scores as GM score ≤ 10.

* Excluded case: Histological placental abruption without anamnestic PSE
